# Reduced impact of nonverbal cues during integration of verbal and nonverbal emotional information in adults with high-functioning autism

**DOI:** 10.3389/fpsyt.2022.1069028

**Published:** 2023-01-09

**Authors:** Michael Alexander Pelzl, Gabrielle Travers-Podmaniczky, Carolin Brück, Heike Jacob, Jonatan Hoffmann, Anne Martinelli, Lea Hölz, Dominik Wabersich-Flad, Dirk Wildgruber

**Affiliations:** ^1^Department of Psychiatry and Psychotherapy, Tübingen Center for Mental Health, University of Tübingen, Tübingen, Germany; ^2^School of Psychology, Fresenius University of Applied Sciences, Frankfurt, Germany

**Keywords:** autism, social cognition, interaction, facial expression, emotional prosody, nonverbal, verbal, high-functioning

## Abstract

**Background:**

When receiving mismatching nonverbal and verbal signals, most people tend to base their judgment regarding the current emotional state of others primarily on nonverbal information. However, individuals with high-functioning autism (HFA) have been described as having difficulties interpreting nonverbal signals. Recognizing emotional states correctly is highly important for successful social interaction. Alterations in perception of nonverbal emotional cues presumably contribute to misunderstanding and impairments in social interactions.

**Methods:**

To evaluate autism-specific differences in the relative impact of nonverbal and verbal cues, 18 adults with HFA (14 male and four female subjects, mean age 36.7 years (SD 11.4) and 18 age, gender and IQ-matched typically developed controls [14 m/4 f, mean age 36.4 years (SD 12.2)] rated the emotional state of speakers in video sequences with partly mismatching emotional signals. Standardized linear regression coefficients were calculated as a measure of the reliance on the nonverbal and verbal components of the videos for each participant. Regression coefficients were then compared between groups to test the hypothesis that autistic adults base their social evaluations less strongly on nonverbal information. Further exploratory analyses were performed for differences in valence ratings and response times.

**Results:**

Compared to the typically developed control group, nonverbal cue reliance was reduced in adults with high-functioning autism [*t*(23.14) = −2.44, *p* = 0.01 (one-sided)]. Furthermore, the exploratory analyses showed a tendency to avoid extreme answers in the HFA group, observable *via* less positive as well as less negative valence ratings in response to emotional expressions of increasingly strong valence. In addition, response time was generally longer in HFA compared to the control group [*F* (1, 33) = 10.65, *p* = 0.004].

**Conclusion:**

These findings suggest reduced impact of nonverbal cues and longer processing times in the analysis of multimodal emotional information, which may be associated with a subjectively lower relevance of this information and/or more processing difficulties for people with HFA. The less extreme answering tendency may indicate a lower sensitivity for nonverbal valence expression in HFA or result from a tendency to avoid incorrect answers when confronted with greater uncertainty in interpreting emotional states.

## Introduction

In daily interpersonal interactions, humans process many verbal and nonverbal cues to monitor the emotional states, intentions, and attitudes of their interaction partners (1). In some cases, a mismatch between the verbal and nonverbal components of emotional cues may occur, for example, when there is social pressure to not show certain feelings or if irony is being expressed. Several studies on this topic have reported a general tendency in healthy participants to weigh nonverbal information more than verbal information when confronted with a mismatch between the two [e.g., (2–8)]. This effect, referred to as “nonverbal dominance,” may be due to nonverbal expressions being considered more difficult to fake and therefore more likely to reveal a speaker’s true emotional state (6). The initial studies on nonverbal dominance by Mehrabian et al. (2), regarded as sensational findings, were often misinterpreted in the media, being presented as evidence that 90% of all information is expressed non-verbally. Scientifically, these results were additionally challenged on methodical grounds [e.g., (9)], but the main findings have been replicated in numerous studies, with individual nonverbal dominance scores ranging from 55 to 100% with a mean value of approximately 90% (4, 5) referring to the relative impact on estimation of the current emotional state of others. Nevertheless, partly contradictory results regarding the respective impact of various nonverbal and verbal communication channels remain. An early review summarizing these contradictory results suggests the greatest reliance on visual information, followed by tone of voice (10). Furthermore, issues regarding the external validity of prior work have been raised, with improvement suggestions including the use of ecologically valid stimuli to allow for generalization to everyday, naturalistic interpersonal communication.

Due to the respective relevance of both verbal and nonverbal cues, it is conceivable that individuals with difficulties decoding one or both channels may show altered reliance on each during multi-channel communication. Such altered dependencies on verbal or nonverbal cues may in turn influence social interaction experiences in everyday life. Indeed, several studies indicate difficulties in processing nonverbal signals as a key factor for many of the social issues displayed, for example, in high-functioning autism (HFA), e.g., (11–13). Autism spectrum disorder (ASD) is a cluster of developmental disorders characterized by difficulties in the processing and display of nonverbal signals, special interests and repetitive behavioral patterns, leading to impairments of social interactions and the formation of social relationships (11). The “spectrum” nature of ASD lies in the large variety of symptom severity within this group. Individuals in the subgroups of high-functioning early childhood autism (F84.0) and Asperger syndrome (F84.5) are among those with the least severe impairments in terms of developmental and intellectual abilities [ref. to F84.0 and 84.5 criteria in the German ICD-10 (14)]. In addition, they are more likely to be diagnosed in adulthood, as more severe forms of ASD tend to be diagnosed in childhood. The aim of the current study was to investigate nonverbal dominance in adults with HFA compared to a typically developed control group, in the absence of differences in intellectual ability.

Previously, it has been reported that individuals on the autism spectrum have difficulties in identifying and interpreting nonverbal cues, with the possible consequence that they pay more attention to the seemingly easier-to-interpret and more rational verbal component of a message as a compensation strategy (15). To test the hypothesis of decreased reliance on nonverbal cues in HFA, the current study combines mismatching verbal and nonverbal information, which have not been implemented to test nonverbal dominance in ASD to date. In addition, a recent review (13) highlighted that emotion recognition impairment based on only prosodic information in ASD might partially be overestimated due to publication bias. The same problem could be present in published data regarding other nonverbal cues, such that the authors strongly recommend more multimodal and differentiated studies to tackle the many inconclusive results. Therefore, this study additionally implements nonverbal information expressed by two nonverbal modalities—emotional facial expressions and affective prosody—simultaneously, combined with either matching or mismatching verbal information about the speaker’s current emotional state. Thereby, the current paradigm creates a situation in which it is important to weigh both components for rating the emotional state of a speaker from an outside perspective. While framed within the context of a study, this paradigm provides an ecologically valid model for real-life everyday interactions.

Specifically, the current study addresses the following hypotheses using stimuli comprising verbal (statements about the current affective state of the speaker) and nonverbal (facial and vocal expressions) emotional cues:

**Hypothesis 1a**: Nonverbal cues have a lower influence on the rating of the current emotional state of the speaker for people with HFA compared to typically developed controls.

**Hypothesis 1b**: Verbal cues have a higher influence on the rating of the current emotional state of the speaker for people with HFA compared to typically developed controls.

Additionally, explorative analyses were carried out to evaluate group differences regarding valence ratings and response times.

## Materials and methods

### Participants

Individuals on the autism spectrum were diagnosed with high-functioning early childhood autism (F84.0) or Asperger syndrome (F84.5) according to the ICD-10 criteria at the special outpatient consultation service for adults with autism-spectrum disorders of the University Hospital Department of Psychiatry and Psychotherapy Tübingen. None of the participants in the HFA group had another psychiatric diagnosis. Four participants received psychiatric medication (*n* = 1 each: combination of Amitriptyline/Venlafaxine, Agomelatine, Paroxetine, and combination of Zyprexa/Venlafaxine). Diagnoses were confirmed by trained psychiatrists and psychologists after intense clinical examination, including anamnesis, and evaluation of interactional behavior and structured questionnaires. Along with the self-report Autism-spectrum Quotient (AQ), validated to quantify autistic traits and empathy [for more details see, e.g., (16, 17)], at least one relative able to report first-hand about the participant’s behavior during the first decade of life completed the Social Responsiveness Scale (SRS), the Social Communication Questionnaire (SCQ/FSK), and the Marburg Rating Scale for Asperger’s Syndrome (MBAS).

The control group (CON) was matched in sex, age, and educational background (see Table 1). Autistic traits were screened in the control group with the AQ questionnaire to confirm that they were not on the autism spectrum themselves. According to the suggestions by Woodbury-Smith et al. (18), scores above 26 were used as an exclusion criterion. Both groups completed the multiple-choice vocabulary intelligence test [MWT-B; (for more details see (19)] as a measure of IQ, along with the Beck Depression Inventory [BDI; (20)] to assess and control for depressive symptoms. All participants were informed in detail about the objectives and procedures involved in this study and signed a written consent form before taking part. The study was approved by the Ethics Committee of the University of Tübingen and fulfills the ethical standards of experiments with humans declared in the revised form of the Helsiniki Declaration from 2008.

**TABLE 1:** Demographic information for both groups and group comparisons.

Group, *N* (Male/Female)	CON, *n* = 18 (14 M/4 F)			HFA, *n* = 18 (14 M/4 F)			*P* (*t*-test)
	***M***	***SD***	**Range**	***M***	***SD***	**Range**	
Age [years]	36.41	12.18	22–62	36.72	11.36	23–57	0.938
Years of education	11.78	1.59	9–13	11.72	1.78	9–13	0.922
IQ	117.47	18.45	94–145	120.31	18.62	92–145	0.663
BDI	2.00	2.54	0–10	13.78	10.45	3–37	<0.001
AQ	11.83	3.11	7–20	37.24	7.73	19–44	<0.001

Demographics table for both groups with between group demographic statistics. Groups were matched in age, sex, and education. Statistically significant differences exist in BDI and AQ scores. CON, typically developed control group; HFA, adults with highly functioning autism.

### Procedure and stimulus material

Participants were instructed to rate the emotional state of a speaker in 120 videos. The persons presented in the videos were all professional actors (five males and five females). The videos showed the faces of the actors while speaking one of six short sentences with high frequencies of use in everyday life. At the verbal level, each sentence expressed information about the current emotional state of the speaker (very positive, positive, neutral, negative, and very negative). At the nonverbal level, facial expressions, and congruent vocal modulations (affective prosody) expressed one of five different emotional states. These included the same five levels as the verbal cues, with angry as the negative and happy as the positive displayed emotion. Nonverbal (facial and vocal) and verbal cues were presented simultaneously, such that the participant had several verbal and nonverbal cues to base their decision regarding the emotional state of the speaker on.

The combination of verbal and nonverbal cues was varied systematically and presented in every possible combination (see Table 2), creating 48 “matching” combinations (verbal and nonverbal indicating the same valence direction, e.g., both positive, both negative, both neutral) and 72 “mismatching” combinations [any valence differences, e.g., negative verbal sentence with non-negative (neutral or positive) nonverbal facial and vocal expression]. The two nonverbal cues of facial expression and prosodic voice modulation were always matched to one another. In a prior study (4), the nonverbal cues were tested for authenticity. Only stimuli characterized by average authenticity ratings of at least 4 points on a 9-point scale were included in the final stimulus set. Moreover, the emotional valence of the verbal and nonverbal component of each stimulus were evaluated separately. To this end the verbal components were rated using written sentences (not containing any nonverbal cues) and the nonverbal components were evaluated using only the sentences with neutral content [for more details on the reference ratings and stimulus material please refer to Jacob et al. (4, 5)], where the full procedure of the recording, processing and evaluation of the videos is described in more detail.

**TABLE 2:** Stimulus material for all combinations of verbal and nonverbal expression.

Verbal expression	Nonverbal expression					
	**Very positive**	**Positive**	**Neutral**	**Negative**	**Very negative**	**Total**
	**+ +**	**+**		**–**	**– –**	
**(Very) positive**						
+ + Ich fuehle mich grossartig (I feel great)	5	3	6	3	3	20
+ Ich fuehle mich gut (I feel good)	3	5	6	3	3	20
**Neutral**						
Ich bin ruhig (I am calm)	3	3	8	3	3	20
Ich bin etwas aufgeregt (I am slightly excited)	3	3	8	3	3	20
**(Very) negative**						
− Ich fuehle mich unwohl (I feel uncomfortable)	3	3	6	5	3	20
− − Ich fuehle mich erbaermlich (I feel awful)	3	3	6	3	5	20
Total	20	20	40	20	20	120

Specific sentences for all valence levels of verbal expressions in German (and translation to English) shown vertically in the first column. Nonverbal valence levels with their symbols shown in the first and second rows. Table shows the total number of stimuli for each combination of verbal and nonverbal cues.

Participants were instructed to rate the current emotional state of the video character from a 3rd person perspective and therefore rely on their overall impression by taking all available cues into consideration. Their rating was to be made on a scale of four different possibilities (very negative, negative, positive, or very positive). The neutral possibility was left out to prevent a central tendency in the answers. Participants were told to base their decision on their first impression and answer as quickly as possible. They could enter their answer while the video was playing and up to 5 s after the end of the video. As feedback, the program highlighted the participant’s response on the screen without rating the correctness of the answer to confirm that the answer was registered before the next sequence. The stimuli were arranged in two blocks with a 12-min duration each, offering a short break in between. Within each block, the order of stimuli was randomized; half of the participants started with block one, while the other half started with the second block. Before the actual experiment, a trial session with 10 additional videos showing each of the actors allowed participants to become familiar with the procedure. These sequences were not used again in the actual experiment.

Stimuli were presented using the software “Presentation” (Neurobehavioral Systems Inc., Albany, CA USA) on a standard computer with a 17-inch flat screen monitor. For the sound output, binaural headphones were used (Sennheiser HD 515; Sennheiser electronic GmbH & Co. KG, Wedemark-Wennebostel, Germany) with volume levels as preferred by the subjects. A Cedrus RB-730 response pad (Cedrus Corporation, San Pedro, CA, USA) allowed participants to submit their valence rating using four buttons with the rating options + + (very positive), + (positive), − (negative), and − − (very negative). For half of the participants, the order of the buttons on the answer pad was reversed to avoid an effect of the positioning of the answers. For the sound output, binaural headphones (Sennheiser HD 515; Sennheiser electronic GmbH & Co. KG, Wedemark-Wennebostel, Germany) with volume levels as preferred by the subjects were used. For statistical analysis, numeric values from 1 to 4 were assigned to each valence rating with 1 = “very negative” and 4 = “very positive.”

### Data analysis

#### Regression analysis

For quantification of the respective impact of nonverbal and verbal cues on the valence ratings of each participant, individual linear regression analyses were calculated. Hereby, the normative nonverbal and verbal valence ratings of the stimulus selection study [(4, 5), see Stimulus Material above] were used as the predictor variable, and the participant’s individual rating of the emotional state of the stimuli was used as the dependent variable. Thus, per participant, two standardized regression coefficients were calculated. These estimated how well the participant’s overall rating of the emotional state for each stimulus could be predicted by the mean emotional valence assigned purely to the (a) nonverbal component (Beta-nonverbal, β_*NV*_) and (b) verbal component (Beta-verbal, β_V_) by the normative sample. The analysis was performed for each participant individually.

#### Group comparisons

The resulting standardized regression coefficients were then tested for group differences with a one-sided heteroscedastic *T*-test for independent groups (HFA vs. CON) with a significance level set to 2.5% (0.025) corrected with Bonferroni correction for two tests. In an additional confirmatory analysis, an ANCOVA (analysis of covariance) including the BDI as a covariate was carried out to evaluate if the results remained significant after controlling for the influence of depressive symptoms. Linear regression and correlation analyses were performed to evaluate further relationships between IQ and AQ on the dependent variables. To operationalize the tendency toward extreme valence ratings, the standard deviation of each participant’s individual valence ratings was included as the dependent variable in multiple linear regression analyses, and associations with the predictor variables BDI and AQ were calculated. Within the further explorative analysis, two mixed-model analyses of variance (ANOVA) were used to investigate overall group differences in valence ratings and response times as well as interaction effects of the verbal and nonverbal components using the verbal valence (five different levels) and nonverbal valence (five different levels) as within-subject factors and the group (HFA vs. CON) as a between-subject factor. The ANOVA *p*-values were corrected using the Geiser–Greenhouse correction method. *Post hoc* two-sided heteroscedastic *t*-tests were performed when necessary for proper interpretation of interaction effects. To avoid reducing sensitivity and due to the explorative character of this analysis, *post hoc* comparisons were not corrected. IBM SPSS Statistics Version 28 was used for all statistical analyses (IBM Corp., Armonk, NY, USA).

## Results

### Impact of nonverbal and verbal cues on judgments of current emotional states

**FIGURE 1:**
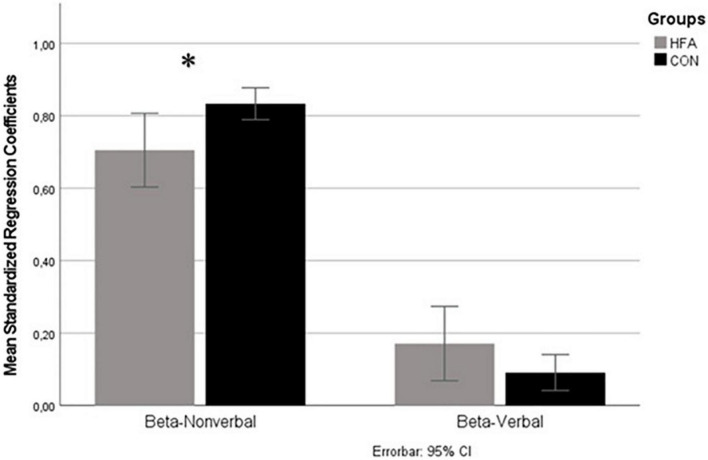
Standardized regression coefficients quantifying the impact of nonverbal (beta-nonverbal) and verbal (beta-verbal) cues on rating of affective states. Means of Beta-coefficients of regression analysis on the *y*-axis, sources of information (verbal and nonverbal) on *x*-axis. Gray column represents high-functioning autism (HFA) group, black column typically developed control group. Errorbars indicate 95%-confidence interval. Significant differences are indicated by asterisks (level of significance *<0.05). A significant difference in the nonverbal condition was found, with a reduced impact of nonverbal cues in the HFA group as compared to the control group (CON) group. The difference in the impact of verbal cues showed a tendency to weight these more strongly in the HFA group at trend level.

The comparison of standardized regression coefficients between the two groups resulted in a significant difference in the impact of nonverbal cues on the ratings of emotional states [Beta-nonverbal β_*NV–CON*_ = 0.83, β_NV–HFA_ = 0.71, *t*(23.14) = −2.44; *p* = 0.01, significance set to <0.025 with Bonferroni correction for two tests; see Figure 1], revealing a reduced impact of nonverbal signals in the HFA group with a large effect size (Cohen’s d = 0.81). In the case of the regression coefficient for the impact of verbal cues (Beta-verbal, β_V_), no significant difference between the groups was detected, but a trend was observed toward HFA focusing more on verbal expression (mean β_*V–CON*_ = 0.09, β_V–HFA_ = 0.17; *p* = 0.06; see Figure 1). The estimated effect size was medium (Cohen’s d = 0.49). The confirmatory analysis of covariance (ANCOVA) also showed a significant group difference for the Beta-nonverbal after correcting for BDI [*F* (1, 32) = 4.01, *p* = 0.027]. Regression analysis of IQ-values with Beta nonverbal did not reach significance while AQ was significantly negatively associated to Beta-nonverbal (β = −0.441, *p* = 0.009).

### Explorative analysis of interactions between the impact of nonverbal cues, verbal cues, and group differences on judgments of emotional states

To analyze possible interactions effects, an ANOVA was conducted with the verbal and nonverbal expression levels as within-subject variables and group as the between-subject variable. This resulted in significant main effects of verbal [*F* (1.703, 56.214) = 19.582, *p* < 0.001] and nonverbal expression level [*F* (1.484, 48.956) = 380.260, *p* < 0.001], and no significant main effect of group. Significant interactions were found between verbal and nonverbal expression levels [*F* (5.563, 183.578) = 15.127, *p* < 0.001], as well as between group and nonverbal expression level [*F* (1.48, 49) = 8.06, *p* = 0.002], but not between group and verbal expression level.

**FIGURE 2:**
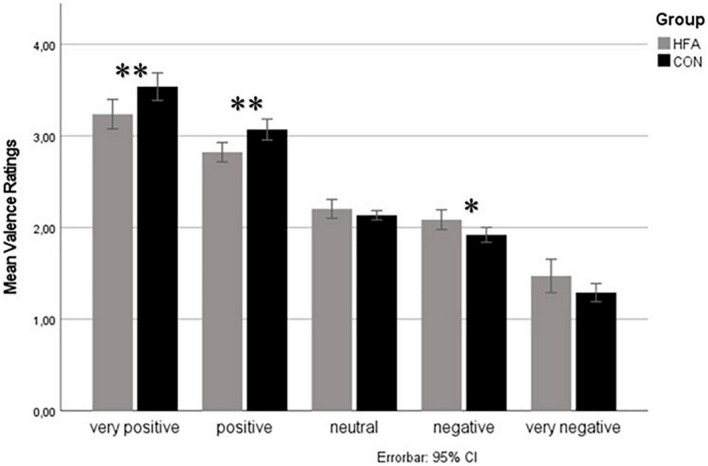
Valence ratings for different nonverbal valence levels. Mean valence ratings for different levels of nonverbal valence on the *y*-axis. The gray column represents the high-functioning autism (HFA) group, and the black column the typically developed control group. Errorbars indicate 95%-confidence intervals. Significant group differences are indicated by asterisks (level of significance *<0.05, ^**^<0.01) Significant differences between the groups in “very positive”, “positive”, and “negative” valence levels. Valence ratings of the HFA group compared to controls are less extremely/more neutrally directed in every condition.

To better characterize the group × nonverbal valence level interaction, *post hoc* two-sided heteroscedastic *t*-tests were calculated for rating differences between groups for each of the five nonverbal valence levels. Hereby, significant group differences were found in positive (happy) nonverbal expression, with the adults with HFA rating the expression less positive compared to controls (mean valence rating CON = 3.1, HFA = 2.8, *p* = 0.002) and in the very positive (happy) condition (mean valence rating CON = 3.5, HFA = 3.2, *p* = 0.007). The negative (angry) nonverbal condition was also significantly different, with the adults with HFA rating the expression less negatively (mean valence ratings CON = 1.9, HFA = 2.1, *p* = 0.013). No differences were found in the very negative (angry) and neutral nonverbal expression conditions (see Figure 2).

The exploratory regression analysis revealed a significant association of the tendency toward less extreme valence ratings and the severity of autistic symptoms (β = - 0.508, *p* = 0.046), whereas no association with the severity of depressive symptoms was observed (β = 0.041, *p* = 0.87).

### Explorative analysis of interactions between the impact of nonverbal cues, verbal cues and group differences on response times

**FIGURE 3:**
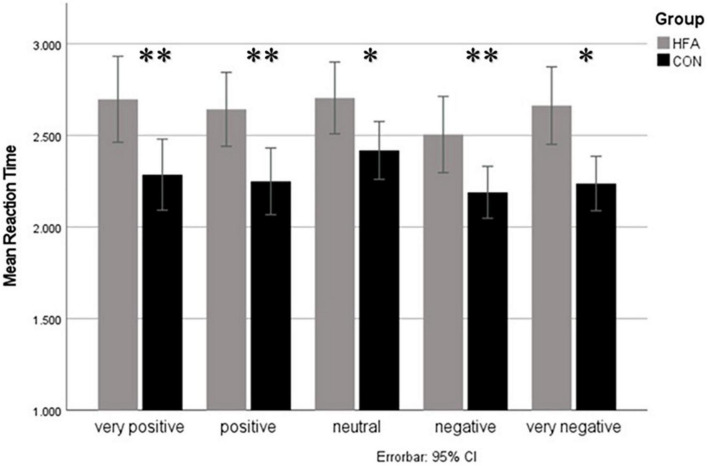
Response times for different nonverbal valence levels. Mean response time (in ms) for different levels of nonverbal valence on the *y*-axis. The gray column represents the high-functioning autism (HFA) group, and the black column the typically developed control group. Errorbars indicate 95%-confidence intervals. Significant group differences are indicated by asterisks (level of significance *<0.05, ^**^<0.01). Significantly slower responses of the HFA group compared to the control group were observed in every valence level.

A second 5 × 5 × 2 ANOVA on mean response times (in ms) for each valence level of verbal and nonverbal cues as a within-subject variable and group as the between-subjects variable demonstrated a significant main effect of the group [*F* (1, 33) = 10.65, *p* = 0.004]., indicating significantly slower response times in the HFA compared to the CON group independently of the cue valence level. Other main effects were found for nonverbal valence level [*F* (3.12, 99.78) = 5.92, *p* < 0.001] and verbal valence level [*F* (3.60, 118.78) = 30.32, *p* < 0.001] as well as the interaction of both [*F* (5.56, 183.58) = 15.13, *p* < 0.001]. The interactions between group and verbal as well as group and nonverbal valence level did not reach significance. *Post hoc* analyses of these effects revealed that the only significant difference over all valence levels between the groups was the generally slower response time in the HFA group, which was nearly equal over all stimulus conditions with a large effect size (Cohen’s d = −1.02) (see Figure 3). This result remained significant after correcting for the group difference in depressive symptoms by including BDI scores within an ANCOVA approach (no significant interaction effect of BDI [*F* (1, 32) = 0.60, *p* = 0.809], preserved significant main effect of group [*F* (1, 32) = 6.65, *p* = 0.015]. Multiple linear regression of the IQ and AQ with mean response times showed a significant association only for the AQ (β = 0.56, *p* = 0.001).

## Discussion

To our knowledge, the current study is the first to measure the relative influence of verbal and nonverbal cues on judgments of the current emotional state of the speaker in individuals with HFA. The results show a significantly reduced impact of nonverbal cues and a non-significant tendency for a stronger impact of verbal cues as compared to typically developed individuals.

The significantly lower Beta-nonverbal coefficient in the HFA group (as a mathematical representation of the reduced impact on nonverbal cues when rating the current emotional state of a speaker) can be interpreted as evidence for the previously suggested difficulties in processing nonverbal signals such as prosody and facial expression (12, 15, 21). The mechanism by which such difficulties may lead to a shift in nonverbal reliance may be multifold. It is conceivable that adults with HFA receive social feedback indicating that their interpretation of nonverbal information is unreliable. Alternatively, or as a consequence, a subjective and adverse feeling of uncertainty may occur when decoding nonverbal cues. Either mechanism could result in a conscious or unconscious shift in attentional focus away from nonverbal cues, and a prioritization of less equivocal information, such as the semantic content of verbal cues. Thus, a compensatory overreliance on verbal cues has been proposed by some authors, e.g., (15). In the current study, while evidence for less reliance on nonverbal cues was found (Cohen’s d = 0.81), a compensatory overreliance on the verbal content, represented by a higher Beta-verbal, remained at a statistical trend level. The reason for this might be the relatively low number of participants, which may have been too few to reach significance for the observed medium effect size (Cohen’s d = 0.49). In further research, it would be interesting to see if this result can be replicated in other studies and experimental setups and if the Beta-verbal reaches significance with a larger number of participants. To allow for a sample size estimation for further studies we calculated effect sizes wherever appropriate. Based on the medium effect size of 0.494 for the increased impact of verbal cues in adults with HFA, for example, 104 participants (52 per group) would be required to expect a significant difference as compared to typically developed controls [one-sided *t*-test, *p* < 0.05, power 0.80)].

A particularly interesting finding in the explorative ANOVA was the interaction of the group and the nonverbal cues. The *post hoc* analysis of this interaction showed a tendency toward neutral ratings for nonverbal cues in the HFA group (see Figure 2). On the positive end of the valence spectrum, the significantly less positive ratings in the “positive” and “very positive” stimulus conditions in HFA compared to the control group could be interpreted as support for a negativity bias (22). Eack et al. (22) described this bias in individuals with HFA, in which happy faces were rated as neutral, while neutral faces were rated as angry or sad. However, in the current study, no negativity bias was observed on the opposite end of the valence spectrum. Instead, the HFA group rated “negative” stimuli as significantly less negative than the control group. This pattern was observed at a trend level in the “very negative” condition, while there was no difference between groups in the “neutral” condition. Thus, rather than a negativity bias, the current analysis shows a tendency for adults with HFA to rate both negative and positive nonverbal stimuli as less extreme than the control group. A simple explanation of this phenomenon could be that autistic persons may feel overwhelmed when required to give an answer to an unsolved problem under time pressure, leading to random or more neutral answers as signs of uncertainty (13). In addition, perception deficits may lead to incorrect assessments, with deviations that can only occur in a negative direction for extremely positive stimuli and only in a positive direction for extremely negative stimuli. In total, such deviations would result in a more neutral answering tendency. However, studies also postulate a lower sensitivity to emotional expression intensity in HFA, which could also lead to a lower valence rating in both extreme directions and a tendency to more neutral ratings (23). Similarly, the lower prioritization of nonverbal information as discussed above might also result in a reduction in the perceived intensity of the stimuli, i.e., a systematic bias in the direction of neutral, which is stronger the more intense the stimulus. The pattern found here has been reported in previous studies. Using happy and sad prosodic cues, Gebauer et al. (24) similarly reported the positive emotion “happy” to be rated less positively in HFA and the negative emotion “sad” to be rated less negatively, in the absence of additional verbal emotional information. Clark et al. (25) also found evidence for a “less positive” bias (more negative valence ratings of positive expressions) when processing nonverbal prosodic information, but only when stimuli were presented shortly and spontaneously, as was also done in the current study. The current study thus extends these findings by demonstrating this effect to be independent of matching or mismatching simultaneously presented verbal emotional information. In addition, the tendency to less extreme answering was found in HFA despite the presence of congruent nonverbal emotional information over multiple channels (emotional facial and prosodic cues). Thus, the current study indicates a robustness of this response tendency even in situations in which all social information sources indicate the same emotional state of the speaker. It should be noted, however, that the instruction to respond quickly and/or spontaneously may augment deviant answering tendencies in individuals with HFA (25), which may explain contradictory results in the literature.

Another overall finding in our study, which is already discussed in the literature on ASD (26), was the slower response time in the HFA group than in the control group in all conditions (Cohen’s d = −1.02). This may lie in higher conscientiousness traits in individuals with HFA, who therefore work more slowly to avoid mistakes (22). In contrast to our study, Doody and Bull (26) found response times to be slower only in identifying emotional body postures, while accuracy and response times when rating prosodic expressions did not differ from the control group. Doody and Bull (26) came to the conclusion that persons with ASD take longer to process nonverbal body posture information but can be as accurate in identifying it if given more time. This could have an impact on training programs for the social competence of people with autism spectrum disorders, which could focus on verbalizing the need for more time or training accelerated processing to facilitate social interaction. An alternative interpretation of the observed slower response times when rating valence could be that some adults with ASD have learned strategies to identify nonverbal cues and take them into consideration, but that this conscious integration takes longer than the more intuitive valence rating process presumably implemented by typically developed individuals (27). Even without training, a more explicit processing style has been reported in ASD (28). One reason why Doody and Bull (26) did not find longer response times to prosodic cues could be the unimodal presentation of nonverbal stimuli, compared to the simultaneous presentation of emotional valence information *via* two nonverbal channels in the current study. The explicit processing of nonverbal information in ASD, as opposed to the more intuitive, implicit processing of typically developed adults, could be more time-consuming if more information has to be processed and is presented simultaneously.

One of the strengths of this study is that the stimuli (verbal and nonverbal information displayed simultaneously, prosody and facial information always matching, verbal information partly contradictory) resemble realistic ways of emotion expression that could occur in everyday situations. To our knowledge, no other study has integrated so many aspects that are relevant in everyday interactions in one paradigm, while measuring the relative influence of verbal and nonverbal cues on valence rating. One of the earliest reviews on this topic pointed out that only the results of studies implementing ecologically valid paradigms should be used for generalization (10). In addition, the paradigm used here has been validated and successfully used in other studies on different disorders, e.g., borderline personality disorder (29) and schizophrenia (7).

Limitations of the study include the relatively small number of participants. Our results should be seen as first findings, that still need validation in larger studies and can help to estimate case numbers for similar studies or inspire other researchers to adopt new approaches for this interesting topic. Another limitation can be seen in the age range of the professional actors between 30 and 50 years. It is possible that this age range could contribute to varying levels of identification with the speaker, which could facilitate or reduce empathic responding. Another important aspect is that the emotional state of the speaker was of no relevance to the participants in our experimental setup, which can be quite different in everyday life. As with many studies comparing adults with autism to typically developed controls, a further limitation of our study is the potential influence of comorbid disorders (especially depression) and psychiatric medication. While we have attempted to correct for the former through BDI screening and statistical correction, the latter remains difficult to control for, especially in the absence of conclusive studies on the impact of medication on social cognition. As our participants with ASD only consisted of adults with high-functioning early childhood autism (F84.0) or Asperger syndrome (F84.5) according to the ICD-10 criteria, our results are restricted to these subgroups and cannot be generalized to the whole spectrum. One could argue that differences observed in these less severe forms might be even more present in the more affected subgroups, but further studies with the same or a similar paradigm are needed to examine the question of graduated verbal/nonverbal reliance with increasing symptom severity.

An important question for further research, often raised in studies focusing on emotion processing in autism, is whether there is an emotion-specific bias pattern in HFA. One of the largest studies on the processing of nonverbal expression found no disorder specific pattern of emotion confusion in HFA and reported that recognition ability was more dependent on IQ than on the severity of the HFA (30). Autistic adults did not perform significantly worse in recognizing emotions than typically developed controls, except for the emotion “surprise,” which is regarded to be especially difficult to identify by only nonverbal criteria, even for typically developed adults. It might therefore be relevant for further research to replicate our paradigm with different and multimodal emotional expressions at nonverbal and verbal levels and to investigate whether the lower influence of nonverbal expressions on the speaker’s emotional state rating is also present for other positive and negative expressions besides angry and happy. Finally, it might be possible to show an effect on response times and the compensatory higher influence of the verbal component. The latter aspect showed a non-significant trend in the expected direction in the current study, which may become significant with a larger number of participants.

## Data availability statement

The raw data supporting the conclusions of this article will be made available on demand by the authors, without undue reservation.

## Ethics statement

The studies involving human participants were reviewed and approved by the Ethics Committee of the University of Tübingen. The patients/participants provided their written informed consent to participate in this study.

## Author contributions

MP wrote the manuscript and performed the data analysis under supervision of DW. GT-P conducted the experiment and pre-processed the data. JH, LH, AM, and DW assisted in data analysis and reviewed the manuscript. AM contributed to the conceptualization of analyses and interpretation and wrote the manuscript. DW, CB, and HJ designed the experiment. All authors contributed to the article and approved the submitted version.
